# Enhanced superconductivity close to a non-magnetic quantum critical point in electron-doped strontium titanate

**DOI:** 10.1038/s41467-019-08693-1

**Published:** 2019-02-13

**Authors:** Yasuhide Tomioka, Naoki Shirakawa, Keisuke Shibuya, Isao H. Inoue

**Affiliations:** National Institute of Advanced Industrial Science and Technology (AIST), Tsukuba, 305-8565 Japan

## Abstract

Studies on quantum critical points (QCP) have focused on magnetic QCPs to date. Remarkable phenomena such as superconductivity due to avoided criticality have been discovered, but we focus here on the non-magnetic counterpart, i.e., the superconductivity of SrTiO_3_ regarded as being close to a ferroelectric QCP. Here we prepare high-quality Sr_1−*x*_La_*x*_Ti(^16^O_1−*z*_^18^O_*z*_)_3_ single crystals without localisation at low temperatures, which allow us to systematically investigate the La substitution of Sr as an alternative to introducing oxygen vacancies. Analysis of our data based on a theoretical model predicts an appearance of the ferroelectric QCP around 3 × 10^18^ cm^−3^. Because of the QCP, the superconducting dome of Sr_1−*x*_La_*x*_TiO_3_ can be raised upwards. Furthermore, remarkable enhancement of *T*_c_ (~0.6 K) is achieved by ^18^O exchange on the Sr_1−*x*_La_*x*_TiO_3_ crystals. These findings provide a new knob for observing intriguing physics around the ferroelectric QCP.

## Abstract (web summary)

Among its interesting properties, SrTiO_3_ can show both superconductivity and ferroelectric quantum criticality at low temperatures. Tomioka et al. use La and oxygen-isotope doping to tune electron-doped SrTiO_3_ to the critical region and observe enhanced superconductivity, suggesting a link between them.

## Introduction

SrTiO_3_ is one of the most studied transition-metal oxides in the history of condensed matter physics. It is a simple band insulator with a band gap of ~3.3 eV between the Ti 3*d* and O 2*p* bands but exhibits various unique and interesting properties and thus remains at the centre of ardent research^1,2^. SrTiO_3_ undergoes an antiferrodistortive phase transition at ~105 K due to the staggered rotations of TiO_6_ octahedra around the [001] axis. Many studies suggest that this antiferrodistortive transition suppresses the ferroelectric (FE) phase transition^3,4^ at relatively high temperatures, and this scenario is supported by first-principles calculations^5^. However, it remains unclear why the ferroelectricity is suppressed down to very low temperatures (at least 350 mK^6^) despite its phonon structure with polar soft modes remaining. Because of this missing ferroelectricity, a huge static dielectric constant *ε* ~ 24,000 is observed at low temperatures, resulting in a very large effective Bohr radius of ~0.5 µm. Thus, slight carrier doping of even 2 × 10^16^ cm^−3^ leads to the appearance of an extraordinary dilute metallic state^7^. It is generally believed that the missing ferroelectricity, even at low temperatures, is entirely due to quantum fluctuations: i.e., zero-point motion preventing the complete softening of the transverse optic phonons^6,8^. The low-temperature phase is positioned close to a quantum critical point **(**QCP), where different phases compete (such as paraelectric, antiferrodistortive, and FE states with similar energies^9–11^). Near the QCP, any residual interactions may drive the system to a superconducting state^8,12,13^.

Electron-doped SrTiO_3_ is one of the most dilute superconductors^14^ and has been known for more than half a century; however, its mechanism is poorly understood^15,16^. Several theoretical ideas linking the superconductivity of SrTiO_3_ to low-temperature instabilities have been proposed. One typical idea is that a soft phonon mode associated with the antiferrodistortive rotation may play a crucial role in the formation of Cooper pairs^17^. Another representative idea involves quantum fluctuations of the FE ordering^6,18,19^. Systematic experimental studies are needed to clarify the mechanism.

Here, we start by demonstrating an appearance of the superconducting dome; i.e., the dome-shaped evolution of *T*_c_ as a function of *n*, achieved by La substitution of Sr in SrTiO_3_ single crystals. Then, we demonstrate the further enhancement of *T*_c_ by oxygen isotope (^18^O) exchange of the La-substituted SrTiO_3_ single crystals. It should be noted here that La-substituted SrTiO_3_ single crystals have never been studied systematically nor in detail to date. In this work, the optimal *T*_c_ reaches 0.44 K at a carrier density *n* of ~5.9 × 10^19^ cm^−3^. Moreover, for the ^18^O-exchanged La-substituted SrTiO_3_ single crystals, the maximum *T*_c_ is enhanced to 0.55 K at almost the same *n* of ~6.0 × 10^19^ cm^−3^. These values of *n* are slightly lower than *n* ~ 1 × 10^20^ cm^−3^, at which SrTiO_3−*δ*_ exhibits the optimal *T*_c_ (*δ* is the amount of oxygen off-stoichiometry that provides two electrons per *δ* in a formula unit.)^20,21^. These enhancements of *T*_c_ are investigated in this work, and the results suggest a hidden QCP even in a metallic region which may contribute to the *T*_c_ enhancement.

## Results

### Resistivity and superconductivity for La-doped SrTiO_3_

**Fig. 1:**
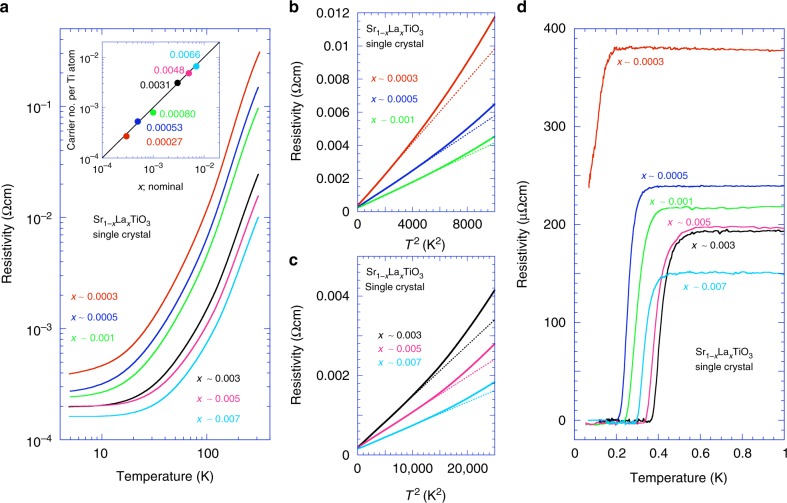
Resistivity and superconductivity of Sr_1-*x*_La_*x*_TiO_3_ single crystals. **a** Temperature dependence of resistivity in Sr_1−*x*_La_*x*_TiO_3_ single crystals with nominal values of *x ~ *0.0003, 0.0005, 0.001, 0.003, 0.005, and 0.007 with which we substituted La for Sr in the raw material. The inset shows that the number of electrons per Ti site determined using the Hall effect measurements was almost identical to the nominal value of *x*. **b**, **c** Resistivity in **a** re-plotted vs. *T*^2^. The straight dotted lines represent the best fits to the data below 40 K. As *x* increases, the slope gradually decreases, and the deviation point of the straight line from the real data shifts to higher temperatures. **d** Resistivity measured using a ^3^He/^4^He dilution refrigerator plotted against *T* below 1 K for the same samples shown in **a**. With increasing La substitution, the residual resistivity decreases monotonously, whereas the value of *T*_c_ increases up to *x* ~ 0.003 and decreases for *x* ~ 0.007

The temperature dependences of the resistivity *ρ* for the single crystals of Sr_1−*x*_La_*x*_TiO_3_ (*x* *~* 0.0003, 0.0005, 0.001, 0.003, 0.005, and 0.007) are presented in Fig. 1a. Each value of *x* is a nominal value, which was used to prepare each sample (see Methods). As shown in the inset of Fig. 1a, each nominal value of *x* is almost identical to the number of carriers per Ti site, which was deduced from Hall effect measurements (see Supplementary Figure 7). It is widely accepted that in the oxygen deficient SrTiO_3−*δ*_, the carrier doping *δ* is directly related to the defect formation on the Ti–O bond of SrTiO_3_, whereas in Sr_1−*x*_La_*x*_TiO_3_, the A-site substitution of the ABO_3_ perovskite-type structure does not directly introduce disorder to the Ti–O conduction paths^22^. At least for the relatively larger carrier-doping region, La substitution would be an ideal method to investigate the carrier-doping-dependent phenomena of SrTiO_3_. On the other hand, the disturbance due to the oxygen defects appears to be less significant in the extremely dilute doping region^7,14,23^, where the La substitution is not easily controlled. It should be noted, in passing, that the ideal thin film of Sr_1−*x*_La_*x*_TiO_3_ fabricated by the molecular beam epitaxy method, the mobility reaches 30,000 cm^2^ V^−1^ s^−1,^^24^. But both this large mobility as well as that of our Sr_1−*x*_La_*x*_TiO_3_ single crystals fit to the general trend of mobility vs. carrier concentration seen in *n*-doped SrTiO_3_^25^ (see Supplementary Note 3).

As apparent from Fig. 1a, the resistivity decreases with increasing La substitution. At 5 K, the resistivity is ~3 × 10^−4^ Ωcm for *x* *~* 0.0005, which changes to ~1.6 × 10^−4^ Ωcm for *x* *~* 0.007. These behaviours appear to reflect that of a conventional metal. The resistivity is plotted as a function of *T*^2^ in Fig. 1b and c. Although the *T*^2^ dependence of the resistivity is in general a representation of a three-dimensional Fermi liquid^26^, it should be noted that the *ρ* ~ *AT*^2^ behaviour of the conventional Fermi liquid requires Umklapp scattering, for which the smallest reciprocal lattice vector must not exceed four times the Fermi wave vector^23^. The smallest carrier density that satisfies this condition is greater than 2 × 10^20^ cm^−3^. Therefore, the carrier density of our Sr_1−*x*_La_*x*_TiO_3_ is too small for Umklapp scattering, indicating that the *ρ* ~ *AT*^2^ behaviour up to high temperatures may be related to other mechanisms^15^. However, it is very intriguing that the behaviour of the coefficient *A* appears to reflect the Lifshitz transition, which is the change of the number of Fermi surfaces that occurs by changing the carrier density. This means the value of *A* is related to the fermiology of SrTiO_3_. It should be noted that this does not underpin the observed *ρ* ~ *AT*^2^ due to the Umklapp scattering in the conventional Fermi liquid. We will discuss this issue later.

Figure 1d shows the temperature dependences of the resistivity below 1 K for the same samples in Fig. 1a measured using a ^3^He/^4^He dilution refrigerator. Notably, no upturn in resistivity was observed upon decreasing the temperature for any of the samples with different *x* values, which differs from the reported weak localisation^27^ or charge Kondo effect observed in Sr_1−*x*_Ca_*x*_TiO_3−*δ*_^28^. The resistive superconducting transitions are clearly observed. In this study, we define the superconducting transition temperature *T*_c_ which gives the mid-point of the resistivity during the resistance drop for the superconductivity as described in the Supplementary Note 4. The values of *T*_c_ increase from 0.26 K for *x* *~* 0.0005 to 0.41 K for *x* *~* 0.003. As *x* increases further, *T*_c_ decreases to 0.34 K for *x* *~* 0.007. All the results are summarised in Table 1.

**Table 1:** Specifications of Sr_1-*x*_La_*x*_TiO_3_ single crystals

*x* (nominal)	*n* (per Ti)	*n* (cm^−3^)	*ρ* (300 K) (Ωcm)	*ρ* (5 K) (Ωcm)	*A* (μΩcmK^−2^)	*μ* (cm^2^V^−1^s^−1^)	*T*_c_ (K)
0.0003	0.00027	4.54 × 10^18^	0.268	3.91 × 10^−4^	0.948	3300	(<0.05)
0.0005	0.00053	8.96 × 10^18^	0.138	2.74 × 10^−4^	0.552	2540	0.26
0.001	0.0008	1.34 × 10^19^	0.0898	2.43 × 10^−4^	0.394	1910	0.29
0.003	0.0031	5.21 × 10^19^	0.0222	1.94 × 10^−4^	0.129	618	0.41
0.005	0.0048	8.01 × 10^19^	0.0145	2.00 × 10^−4^	0.089	389	0.39
0.007	0.0066	1.11 × 10^20^	0.0093	1.62 × 10^−4^	0.059	347	0.34

The nominal value of *x*; the carrier density *n* (per a Ti site and per cm^3^) determined from Hall effect measurements; the resistivity *ρ* at 300 K and 5 K; the coefficient *A* in *ρ*~ *AT*^2^ below 40 K; the Hall mobility *μ* = *n*^−1^e^−1^*ρ*^−1^ at 5 K, where e is an elementary charge; and the superconducting critical temperature *T*_c_ for our Sr_1−*x*_La_*x*_TiO_3_ single crystals

### Comparison between ^18^O-exchanged and ^18^O-free Sr_1-*x*_La_*x*_TiO_3_

**Fig. 2:**
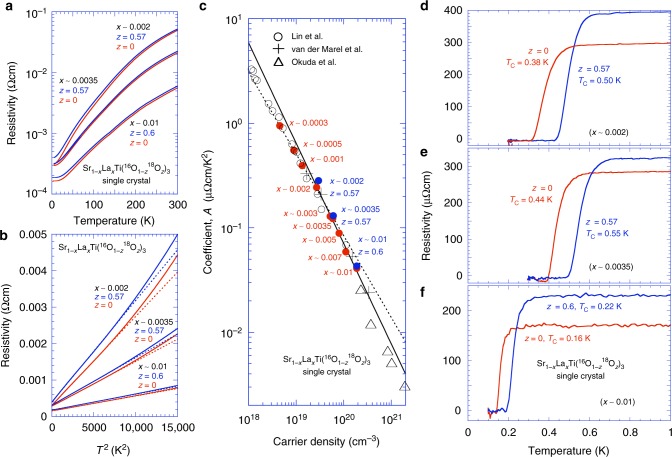
Resistivity and superconductivity of Sr_1−*x*_La_*x*_Ti(^16^O_1−*z*_^18^O_*z*_)_3_ single crystals. **a** Temperature dependence of resistivity for ^18^O-exchanged Sr_1−*x*_La_*x*_Ti(^16^O_1−*z*_^18^O_*z*_)_3_ with (*x*, *z*) = (~0.002, 0.57), (~0.0035, 0.57), and (~0.01, 0.60) (blue lines) compared with that for Sr_1−*x*_La_*x*_TiO_3_ with *x* ~ 0.002, 0.0035, and 0.01 (red lines). **b** Resistivity *ρ* vs. *T*^2^. The dotted lines represent *ρ* ~*AT*^2^ relations to fit the experimental data. The coefficient *A* increases with ^18^O exchange, indicating mass enhancement of the carriers. **c** Logarithmic plot of *A* as a function of carrier density *n* for all our Sr_1−*x*_La_*x*_Ti(^16^O_1−*z*_^18^O_*z*_)_3_ samples in Fig. 1a and **a** with the data in the literature: SrTiO_3–*δ*_^23^ (open circles), SrTi_1−*x*_Nb_*x*_O_3_^15^ (crosses), and Sr_1−*x*_La_*x*_TiO_3_^26^ (triangles). The straight lines are guides to the eye. A two-orders-of-magnitude drop of *A* for increasing *n* is observed, and a kink appears at *n* ~ 4 × 10^19^ cm^−3^ near the critical value for the Lifshitz transition^15,30,31^. **d**–**f** Residual resistivity *ρ*_0_ and superconducting transition temperatures *T*_c_ measured using a ^3^He/^4^He dilution refrigerator below 1 K for the same samples shown in **a**

As described in the Methods section, we prepared two Sr_1−*x*_La_*x*_TiO_3_ single-crystal rods with the same value of *x*, one of which was ^18^O exchanged. For each of the ^18^O-exchanged single crystals, the amount of ^18^O (the value of *z*) was evaluated from the Raman scattering (see Supplementary Figure 1). The temperature dependences of the resistivity for Sr_1−*x*_La_*x*_Ti(^16^O_1−*z*_^18^O_*z*_)_3_ with (*x*, *z*) = (~0.002, 0), (~0.002, 0.57), (~0.0035, 0), (~0.0035, 0.57), (~0.01, 0), and (~0.01, 0.60) are plotted in Fig. 2a. The *z* = 0.57 and *z* = 0.60 samples are denoted hereafter as *z* *=* 0.6 for convenience. Apparent *T*^2^ dependence up to high temperatures was also observed in these samples, as shown in Fig. 2b.

Figure 2c shows the behaviour of the coefficient *A* of *ρ* ~ *AT*^2^ for all the Sr_1−*x*_La_*x*_Ti(^16^O_1−*z*_^18^O_*z*_)_3_ samples in Fig. 1a and Fig. 2a as a function of the carrier density *n*. We deduced the value of *A* by fitting the resistances of all the samples below 40 K (see Fig. 1b and c) to distinguish the low-temperature *T*^2^ behaviour from the high-temperature *T*^3^ behaviour^29^. The obtained value of *A* decreased drastically by approximately two orders of magnitude with increasing carrier density, and a kink was observed near *n* ~ 3.9 × 10^19^ cm^−3^. This value of *n* for the kink is close to the critical carrier density of the Lifshitz transition (*n* ~ 4.4 × 10^19^ cm^−3^ by a band calculation^15^, and ~3 × 10^19^ cm^−3^ by a quantum oscillation measurement^30,31^), where the Fermi energy enters the third *t*_2g_ band. Lin et al. reported a similar kink for *A* in their SrTiO_3−*δ*_ samples when the second *t*_2g_ band starts to be filled^23^. As mentioned above, the coincidence of the kink of *A* and the Lifshitz transition is not easily acceptable. This is because the *ρ* ~ *AT*^2^ without the Umklapp scattering cannot be explained by the conventional Fermi liquid theory, and thus *A* is not necessary to reflect the topology of the Fermi surface. Furthermore, our Sr_1−*x*_La_*x*_TiO_3_ does not show anomaly of *T*_c_ at the Lifshitz transition although the density of states as a function of *n* may have a rapid change at this transition point. These are interesting future problems, which would be a clue for understanding a superconductivity of this system.

Through the ^18^O exchange, the values of *A* become larger than those of the corresponding ^18^O-free ones. If we accept the Fermi liquid-like understanding of *A*, this result indicates that the effective mass of an electron may become larger^32,33^, possibly resulting from enhancement of the electron–phonon interactions. Because of the large electron–phonon interactions, the resistivity in the entire temperature range as well as the superconducting *T*_c_ are enhanced.

Figure 2d–f shows the temperature dependence of the resistivity below 1 K for the same single crystals shown in Fig. 2a. The superconducting transition temperatures of the ^18^O-exchanged and ^18^O-free samples were compared for the *x* *~* 0.002, 0.0035, and 0.01 samples. All the experimentally deduced parameters for Fig. 2 are summarised in Table 2.

**Table 2:** Specifications of ^18^O-exchanged and ^18^O-free single crystals

*x* (nominal)	*n* (per Ti)	*n* (cm^−3^)	*ρ* (300 K) (Ωcm)	*ρ* (5 K) (Ωcm)	*A* (μΩcmK^−2^)	*z*	*T*_c_ (K)
0.002	0.0016	2.70 × 10^19^	0.0495	3.08 × 10^−4^	0.249	0	0.38
	0.0018	2.96 × 10^19^	0.0529	3.99 × 10^−4^	0.281	0.57	0.50
0.0035	0.0036	5.88 × 10^19^	0.0210	2.95 × 10^−4^	0.124	0	0.44
	0.0036	6.04 × 10^19^	0.0224	3.19 × 10^−4^	0.132	0.57	0.55
0.01	0.011	1.88 × 10^19^	0.00599	1.75 × 10^−4^	0.041	0	0.16
	0.011	1.88 × 10^19^	0.00693	2.34 × 10^−4^	0.044	0.60	0.22

The nominal value of *x*, the carrier density *n* (per a Ti site and per cm^3^) determined from Hall effect measurements, the resistivity *ρ* at 300 K and 5 K, the coefficient *A* in *ρ* ~ *AT*^2^ below 40 K, the amount of ^18^O exchange *z* estimated from Raman scattering spectroscopy, and the superconducting critical temperatures *T*_c_ for our Sr_1−*x*_La_*x*_Ti(^16^O_1−*z*_^18^O_*z*_)_3_ single crystals

### Superconducting dome for Sr_1−*x*_La_*x*_Ti(^16^O_1−*z*_^18^O_*z*_)_3_

**Fig. 3:**
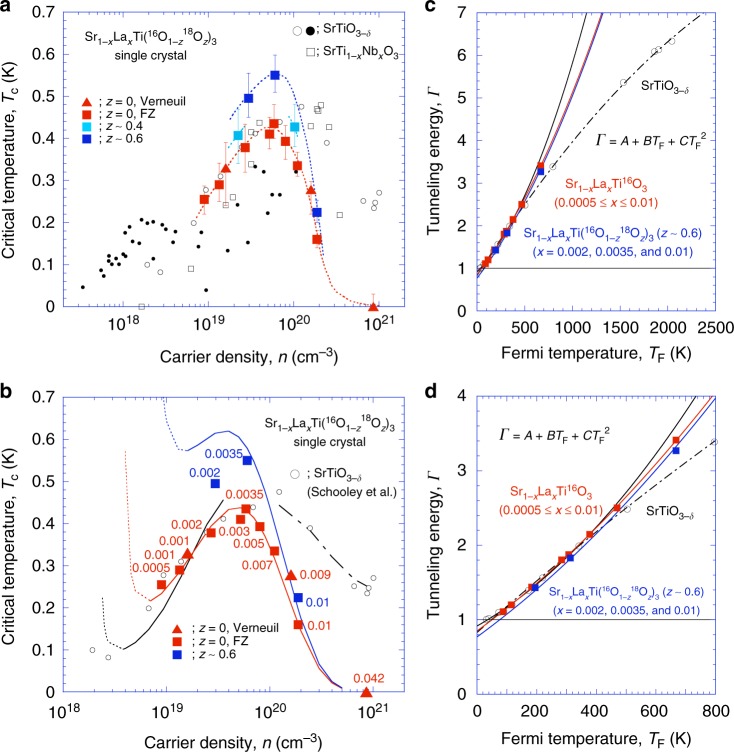
Evolution of the superconducting dome with La substitution and ^18^O exchange. **a** *T*_c_ vs. *n* plot for the Sr_1−*x*_La_*x*_Ti(^16^O_1−*z*_^18^O_*z*_)_3_ single crystals shown in Figs. 1a and 2a. The upper and lower error bars are determined from the onset and end temperatures of the superconductivity (see Supplementary Figure 4). *T*_c_ for SrTiO_3−*δ*_^21,28,31^, SrTi_1−*x*_Nb_*x*_O_3_^31,34^, and La-substituted SrTiO_3_^27^ are also plotted. **b** The lines were obtained from calculations based on the model in ref. ^18^, which are compared with some of the experimental data in **a**. **c** Tunnelling energy *Γ* vs. Fermi temperature *T*_F_ deduced from the experimental values of *T*_c_ and *n* using Supplementary Equations 1 and 2: Sr_1−*x*_La_*x*_TiO_3_ (red squares) and Sr_1−*x*_La_*x*_Ti(^16^O_1−*z*_^18^O_*z*_)_3_ (*z* = 0.6; *x* *~* 0.002, 0.0035, and 0.01) (blue squares) from this study; SrTiO_3−*δ*_ (black circles) from the literature^21^. The experimental data were fitted by the lines of *Γ* = *A* + *BT*_F_ *+* *CT*_F_^2^. From these fitting results for *Γ*, we obtained the *T*_c_ vs. *n* relation represented by the lines in **b**. **d** The blow-up of **c** to emphasise where the *Γ* crosses *Γ* = 1 line

In Fig. 3a, *T*_c_ is plotted as a function of *n* for our Sr_1−*x*_La_*x*_Ti(^16^O_1−*z*_^18^O_*z*_)_3_ single crystals prepared using the floating zone (FZ) method. For the *z* = 0.4 samples, see the Supplementary Note 4. In addition to our data, reported values of *T*_c_ for SrTiO_3−*δ*_^21,28,31^ and SrTi_1−*x*_Nb_*x*_O_3_ single crystals^31,34^ as well as La-substituted SrTiO_3_ single crystals^27^ are also included in the plot; all of these reported samples in the literature were prepared using the Verneuil method. Despite the different crystal growth procedures (FZ or Verneuil), the values of *T*_c_ for all the La-substituted SrTiO_3_ single crystals (*z* = 0) comply with a single superconducting dome. At *x* *~* 0.0035 (*n* ~ 6 × 10^19^ cm^−3^), the dome reached a maximum *T*_c_ of ~0.44 K.

## Discussion

FE fluctuations in SrTiO_3_ with zone-centre soft-mode optical phonons (either longitudinal or transverse) have been considered to play some relevant roles in the mechanism of the superconductivity^16,35–39^. As the FE fluctuations are clearly suppressed with increasing carrier density *n* (as the system becomes more metallic), the concomitant disappearance of the superconductivity for the overdoped region (*n* ≳ 2 × 10^20^ cm^−3^) may be an implication of the superconductivity driven by the ferroelectricity. On the other hand, in the underdoped region (*n* ≲ 1 × 10^20^ cm^−3^), the FE fluctuations are enhanced by the decrease of *n*. However, if *n* is too small, the superconductivity is depressed, as the carrier density is not sufficient to provide robust superconductivity. The formation of the superconducting dome can be explained in this way, though there is room for argument.

What is prominent in Fig. 3a is not only the elucidation of the superconducting dome of Sr_1−*x*_La_*x*_TiO_3_ but also the large enhancement of its *T*_c_ by the ^18^O exchange. As described above, there are many models of the mechanism of superconductivity in SrTiO_3−*δ*_; however, most of these models cannot be simply applied to our experimental data to provide insight into the enhancement by the ^18^O exchange. A recent theoretical approach proposed by Edge et al.^18^ is one of the most tractable approaches, such that we attempted to compare this model to our data as follows. It should be noted here that we do not rule out any other theories which may explain the rise of the superconducting dome.

We calculated the theoretical *T*_c_ vs. *n* curve applying the model in ref. ^18^ to the reported^21^ and our experimental *T*_c_ vs. *n* data. In Fig. 3b, the curves (solid and partially dashed lines) are plotted with the experimental data points. The black line is for SrTiO_3−*δ*_, the red line is for Sr_1−*x*_La_*x*_TiO_3_, and the blue line is for Sr_1−*x*_La_*x*_Ti(^16^O_0.4_^18^O_0.6_)_3_. Details of the calculation steps are provided in Supplementary Note 7. The model explains fairly well the observed superconducting dome of Sr_1−*x*_La_*x*_TiO_3_.

The *T*_c_ vs. *n* lines in Fig. 3b are based on the *Γ* vs. *T*_F_ plots in Fig. 3c. Here, *Γ* is the tunnelling energy of the double-well potential^18^ in analogy with that of magnetic phase transitions, and *T*_F_ is the Fermi energy but we used the value of *T*_F_ deduced from the carrier density by assuming a free electron gas without mass enhancement. The *Γ* vs. *T*_F_ plot was obtained directly from the experimental *T*_c_ vs. *n* data, as described in the Supplementary Note 7. Then, we fit the data using a power expansion of *Γ* in *T*_F_ such as *Γ* = *A* + *BT*_F_ *+* *CT*_F_^2^, where *A*, *B*, and *C* are fitting parameters. The QCP, i.e., the divergence of *λ*, corresponds to the point at which *Γ* = 1. It should be noted that our systematic investigation of La substitution made a reliable fitting possible. Then, it has finally elucidated that *Γ* < 1 below *n* < *n*_c_ in Sr_1−*x*_La_*x*_TiO_3_ as seen in Fig. 3b (the blow-up is plotted in Fig. 3d); this means the model predicts the appearance of a QCP at *n* = *n*_c_ ~ 3 × 10^18^ cm^−3^.

At *n* = *n*_c_, the superconductivity coupling constant *λ* diverges, and *T*_c_ at the QCP becomes unpredictable^18^. Our own numerical calculations following the model produced an exponential enhancement of *T*_c_ at *n* = *n*_c_ (dotted lines in Fig. 3b). It has been suggested that if QCP is above around 1 × 10^18^ cm^−3^, *T*_c_ goes up when the system approaches the QCP^40^. However, the validity of this divergence is controversial. The value of *T*_c_ might be suppressed to zero because the model is not simply applicable in the vicinity of QCP. Unfortunately, for our Sr_1−*x*_La_*x*_TiO_3_ system, it is difficult to explore such an extremely dilute region, where the system actually crosses the QCP. This is an important problem to be clarified by more detailed experimental investigation, such as the application of tensile stress, which is considered to shift the QCP towards the higher doping region leading to a drastic enhancement of *T*_c_ at the QCP^41,42^ or the isostatic pressure to diminish the QCP^40,43^ and suppress *T*_c_.

We further attempted to fit the *Γ* vs. *T*_F_ data of Sr_1−*x*_La_*x*_Ti(^16^O_1−*z*_^18^O_*z*_)_3_ (*z* = 0.6) using *Γ* = *A* + *BT*_F_ *+* *CT*_F_^2^ with the same *B* and *C* values as those for Sr_1−*x*_La_*x*_TiO_3_ (blue dashed line in Fig. 3b). Although this assumption is naive, we do not think that it is unreasonable, because in principle ^18^O exchange does not affect *T*_F_. Because our experimental data are only three points, the fitting was not very good. We understand that *n*_c_ increased; i.e., the QCP shifts towards larger carrier density with the ^18^O exchange. The observed further enhancement of *T*_c_ (blue line in Fig. 3a) can be explained in this way by the shift of QCP.

It should be mentioned that the SrTiO_3−*δ*_ data^21^ in Fig. 3b cannot be simply fitted by the same model in ref. ^18^. It seems that the data might be separated into at least two parts: a smaller carrier-density region below ~5 × 10^19^ cm^−3^, and a larger carrier-density region above it. The fitting of the experimental data in the larger carrier-density region (dash-dotted line in Fig. 3b) suggests there is QCP even for SrTiO_3−*δ*_ at almost the same carrier density as that of Sr_1-*x*_La_*x*_TiO_3_ (see the *Γ* = 1 point of the red solid line and the dash-dotted line in Fig. 3c and d). On the other hand, in the small carrier-density region, although the data of SrTiO_3−*δ*_ are almost identical to those of Sr_1−*x*_La_*x*_TiO_3_, the two additional points in the lowest carrier density make the fitting worse (see the black solid and dotted line). The analysis predicts the QCP may be close to 2 × 10^18^ cm^−3^.

**Fig. 4:**
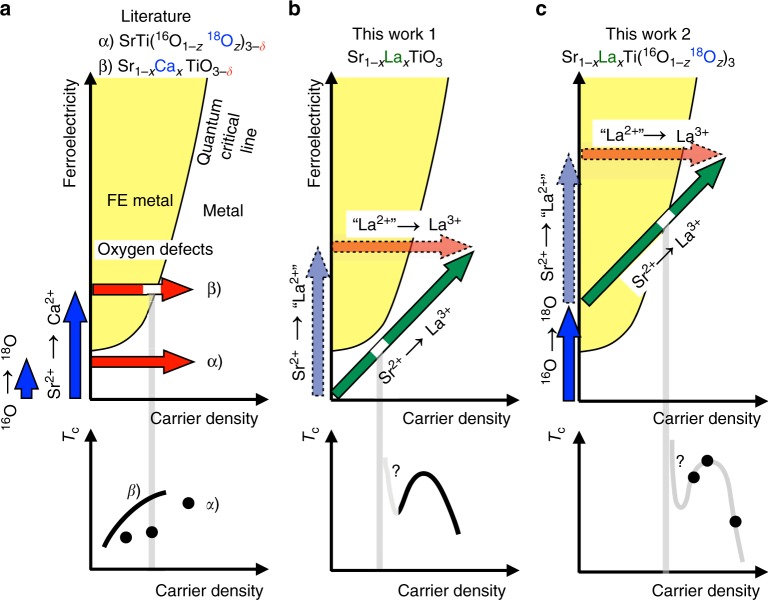
Schematic diagrams of La substitution and ^18^O exchange. Schematic explanation of the evolution of QCP in the metallic state, carrier doping by oxygen defects (red arrows), ^18^O exchange and Ca substitution toward ferroelectricity (blue arrows), as well as the La substitution (green arrow) on the “ferroelectricity” vs. “carrier density” plane. The corresponding *T*_c_ vs. *n* schematics are also shown. **a** α) SrTi(^16^O_1−*z*_^18^O_*z*_)_3−*δ*_^44^ and β) Sr_1−*x*_Ca_*x*_TiO_3−*δ*_^28^, **b** Sr_1−*x*_La_*x*_TiO_3_, and **c** Sr_1−*x*_La_*x*_Ti(^16^O_1−*z*_^18^O_*z*_)_3_. QCP corresponds to the white portion of the arrow at which the behaviour of *T*_c_ becomes unpredictable^18^. However, the shift of QCP towards larger carrier-density region is expected to raise the superconducting dome upwards^18,31,42^

Figure 4 presents schematic diagrams showing the evolution of the FE QCP in the metallic region. The planes are spanned by the horizontal axes of carrier density and the vertical axes of ferroelectricity. The FE state must be rapidly suppressed by the screening due to mobile carriers; however, a certain ordered state was considered to remain even in the metallic phase for very small *n*^18,28^. Hence, the boundary of the ordered FE state is assumed to penetrate into the carrier doped region as shown in Fig. 4^18^.

In the literature, there were two types of investigations of the superconductivity on this plane (Fig. 4a). One is to do ^18^O exchange (blue vertical arrow) and to dope carriers by oxygen-defect creation (red horizontal arrow)^44^. The other is to do Ca^2+^ substitution for Sr^2+^ (blue vertical arrow) and to dope carriers by oxygen-defect creation (red horizontal arrow)^28^. The former did not actually cross the QCP but an enhancement of *T*_c_ was observed. The latter crossed the QCP but there was no anomaly in the *T*_c_ vs. *n* behaviour at QCP, contradicting to the theoretical models^18,42^. However, in these systems, the values of *T*_c_ are relatively large even in the small carrier-density region. There must be a non-negligible contribution of the latent ferroelectricity in the metallic state to the enhancement of the superconductivity.

The La substitution process can be expressed as the diagonal line (Fig. 4b). As discussed in the Supplementary Note 6, if we consider a virtual “La^2+^” substitution as an analogy of Sr_1−*x*_Ba_*x*_TiO_3_ and Sr_1−*x*_Ca_*x*_TiO_3_, it is reasonable to assume that the La substitution process can be decomposed into 1) SrTiO_3_ to Sr_1−*x*_“La^2+^”_*x*_TiO_3_ process (light blue vertical arrow) and 2) Sr_1−*x*_“La^2+^”_*x*_TiO_3_ to Sr_1−*x*_La_*x*_TiO_3_ process (light red horizontal arrow). Therefore, the actual La substitution process is represented by the diagonal green arrow. (This green arrow is not necessary to be the straight one but for simplicity we consider it is a simple straight arrow.) Since there is no experimental evidence, we are not sure the diagonal line crosses the border of the FE metal and the normal metal (i.e., the so-called quantum critical line QCL), but the analysis of our experimental data with the theoretical model^18^ has predicted the appearance of QCP. Although the figure is schematic, we can assume the crossing point (or the point where the diagonal line is in the vicinity of the border line) is located in the low carrier-density region.

The SrTi(^16^O_1−*z*_^18^O_*z*_)_3_ exhibits ferroelectricity at *z* = 0.36 (refs. ^45^. and ^46^). Then, the La substitution process for Sr_1−*x*_La_*x*_Ti(^16^O_1−*z*_^18^O_*z*_)_3_ (*z* > 0.36) corresponds to the green diagonal line in Fig. 4c. This diagonal line is sure to cross the border of the FE metal and the normal metal. The important argument in this schematic diagram is that the crossing point for Sr_1−*x*_La_*x*_Ti(^16^O_1−*z*_^18^O_*z*_)_3_ (*z* > 0.36) should appear at larger *n* than that of Sr_1−*x*_La_*x*_TiO_3_, explaining why *T*_c_ of Sr_1−*x*_La_*x*_Ti(^16^O_1−*z*_^18^O_*z*_)_3_ (*z* > 0.36) is higher than that of Sr_1−*x*_La_*x*_TiO_3_. It is clearly demonstrated that diagonal La substitution *x* in Sr_1−*x*_La_*x*_Ti(^16^O_1−*z*_^18^O_*z*_)_3_ is much more effective for increasing *T*_c_ than the simple horizontal oxygen-defect doping *δ* in SrTi(^16^O_1−*z*_^18^O_*z*_)_3−*δ*_.

In summary, we prepared high-quality Sr_1−*x*_La_*x*_Ti(^16^O_1−*z*_^18^O_*z*_)_3_ single crystals in the low doping region (0.0003 ≲ *x* ≲ 0.01) with oxygen-isotope exchange. La substitution is an ideal method to introduce carriers into SrTiO_3_; our samples do not show tendencies of localisation at low temperatures. The carrier density is too small for Umklapp scattering^23^; however, the *ρ* ~ *AT*^2^ behaviour is seen and the coefficient *A* reflects the topological change of the Fermi surface, suggesting that the Sr_1−*x*_La_*x*_TiO_3_ system cannot be simply understood by a conventional Fermi liquid theory. The superconducting *T*_c_ exhibits a dome shape similar to that of SrTiO_3−*δ*_. The values of *T*_c_ were remarkably enhanced to 0.55 K for Sr_1−*x*_La_*x*_Ti(^16^O_1−*z*_^18^O_*z*_)_3_ (*x* ~ 0.0035 and *z* ~ 0.6). Although the mechanism of the superconductivity in SrTiO_3−*δ*_ and related materials remains a controversial topic, we demonstrated reasonable agreement of our experimental data with the hidden FE QCP model; i.e., the QCP is located in a region where the system is already metallic and will contribute to the *T*_c_ enhancement.

Ferroelectricity is a state of broken inversion symmetry. If we can prepare a sample within the QCP of Sr_1−*x*_La_*x*_Ti(^16^O_1−*z*_^18^O_*z*_)_3_, it may be a novel realisation of noncentrosymmetric superconductivity^47^, which is currently under intensive study, as it may hold mixed-parity pairing mechanisms with topological aspects to their superconducting states. Such a sample will possibly incubate extremely large and highly anisotropic upper critical fields and topologically protected spin currents^48,49^. Our findings will add another page to current research on the FE QCP and associated superconductivity.

## Methods

### Synthesis of Sr_1−*x*_La_*x*_TiO_3_ single crystals

Mixed powders of SrCO_3_, La_2_O_3_, and TiO_2_ in a ratio of 1−*x*:*x*/2:1 were calcined at 500 °C in air for 2–3 h. The calcined powders were sintered at 1000 °C in air for 5 h. Then, the powders were pulverised and formed into a rod with a diameter of approximately 4 mm and length of approximately 60 mm. The rod was fired at 1300 °C–1380 °C for 5 h in a flowing argon gas atmosphere. The crystal growth of Sr_1−*x*_La_*x*_TiO_3_ was performed with a conventional FZ method. In this method, we use a furnace equipped with double hemi-ellipsoidal mirrors coated with gold. Two halogen incandescent lamps were used as heat sources. The crystals were grown in a stream of argon gas, and the growth rate was set at 10–15 mm per hour.

### Synthesis of Sr_1−*x*_La_*x*_Ti(^16^O_1−*z*_^18^O_*z*_)_3_ single crystals

Because the oxygen diffusion in La-substituted single crystals is extremely slow^50^, the oxygen isotope (^18^O) exchange was fulfilled for the mixed powders of SrTiO_3_, La_2_O_3_, and TiO_2_. We first prepared the SrTiO_3_ powders. The mixed powder of SrCO_3_ and TiO_2_ with a molar ratio of 1:1 was calcined at 500 °C for 2–3 h. The calcined powders were sintered at 1280 °C for 30 h in air to synthesise the polycrystalline powders of SrTiO_3_. Then, we mixed the powders of SrTiO_3_, La_2_O_3_, and TiO_2_ in a ratio of 1−*x*:*x*/2:*x* and formed them into a rod with a diameter of 4 mm and length of 60 mm. The rod was sintered at 1000 °C in air for 5 h. The sintered rod was then divided into two rods. In a furnace equipped with double sapphire tubes, one rod was placed in the tube with flowing ^16^O_2_/^18^O_2_ mixed gas atmosphere to exchange the ^16^O atoms with ^18^O ones. The other rod was placed in the other tube in a flowing oxygen (^16^O) atmosphere as a reference. The temperature of the furnace was then increased to 1000 °C to promote the ^16^O/^18^O exchange. The annealing at 1000 °C was continued until the equilibrium concentration of the ^16^O_2_/^18^O_2_ atmosphere was reached. The amount of ^18^O atoms absorbed into the rod was by first estimated from the weight change of the rod and then from the amount of ^18^O_2_ gas in the atmosphere of the furnace, which was determined using a quadrupole mass analyser. These two values matched very well, which indicated that the ^18^O-exchange was sufficiently controlled in our experiments. Both the ^18^O-free and ^18^O-exchanged feed rods were used for the single crystal growth, the procedure for which was the same as described in the previous section. The amount of ^18^O atoms in the grown crystals was finally determined from the frequency shift of the Raman scattering (Supplementary Figure 1).

### Resistivity measurements

The formation of a single crystal was confirmed using the reflection Laue method. Several pieces were cut from the single crystal rod along the [100] direction of the cubic indices. The pieces were further formed into a rectangular shape with the longest dimension parallel to the [100] direction of the cubic indices. The typical dimensions were 0.5 × 0.3 × 7 mm^3^. The d.c. resistivity *ρ* and Hall resistivity *ρ*_H_ for 5 K ≤ *T* ≤ 300 K were measured in a cryostat equipped with a superconducting magnet (Physical Property Measurement System (PPMS), Quantum Design Inc.). The resistivity down to 50 mK was measured using an a.c. resistance bridge (LR700, Linear Research Inc.) in a cryostat with a ^3^He/^4^He dilution refrigerator (*µ* dilution, Taiyo-Toyo Sanso Inc.). The electrodes were prepared using ultrasonic indium soldering. The transport current was injected parallel to the [100] direction of the cubic indices.

## Supplementary information

Supplementary Information

Peer Review File

## Data Availability

The data that support the plots within this paper and other findings of this study are available in figshare with the digital object identifier 10.6084/m9.figshare.7434221 (ref. ^51^). Further data and resources in support of the findings of this study are available from the corresponding authors upon reasonable request.
