# Supplementary material for: Enhanced superconductivity close to a non-magnetic quantum critical point in electron-doped strontium titanate
Source: Nat Commun. 2019 Feb 13;10:738. doi: 10.1038/s41467-019-08693-1 (PMC6374393; doi:10.1038/s41467-019-08693-1)
Supplement: Supplementary file 1 — Supplementary Information [file 41467_2019_8693_MOESM1_ESM.pdf]

Supplementary Information

Enhanced superconductivity close to a non-magnetic quantum critical point in electron-doped strontium titanate

Tomioka *et al.*

Supplementary Note 1: Estimation of the oxygen isotope (^{18}O) content in melt-grown single crystals

Raman spectra were collected at room temperature in the backscattering geometry with unpolarised detection using an InVia Renishaw micro-Raman spectrometer with a 532-nm excitation source. The incident light with a power of 8 mW was focused on the top of the sample along the [100] direction through a 50× objective lens with a laser spot of ~2 μm in diameter. The spectral resolution of the system was ~2 cm^{-1}. The Raman shift was calibrated using a signal of 520 cm^{-1} from a standard silicon wafer.

Supplementary Figure 1(a) shows the frequency dependences of the Raman scattering spectra for both ^{18}O-free ($x \sim 0.002$ and $z = 0$) and ^{18}O-exchanged ($x \sim 0.002$ and $z = 0.57$) single crystals of $Sr_{1-x}La_xTi(^{16}O_{1-z}{}^{18}O_z)_3$. The broad peak in the frequency region from ~600–800 cm^{-1} is a superposition of three second-order Raman peaks (overtones and combinations) located at 629 cm^{-1} (TO_4 + TA, TO_4 + TO_1), 684 cm^{-1} ($2TO_3$), and 727 cm^{-1} (TO_4 + TO_2) [1], where TO_n is the n-th transverse optic phonon mode and TA is the transverse acoustic phonon mode. These peaks are insensitive to temperature change [1] and quite robust during the measurement. Indeed, if we plot each Raman spectrum of the ^{18}O-exchanged sample against the frequency multiplied by a certain factor, the peak in the approximately 600–800 cm^{-1} region coincides with that of the corresponding ^{18}O-free sample almost perfectly. The factor is given by $[\{16(1 - z) + 18z\}/16]^{1/2}$, which means that the frequency is simply modified by the change of the average atomic mass of the oxygen atoms. In Supplementary Figure 1(b), the frequency for the spectrum of the ^{18}O-exchanged sample is multiplied by $[\{16(1 - z) + 18z\}/16]^{1/2} = 1.035$ (for z = 0.57). For the $x \sim 0.0035$ and 0.01 samples, as observed in Supplementary Figures 1(c) and 1(d), the coincidences are also quite good as they are multiplied by factors of 1.035 and 1.037, respectively. The values

correspond to $z = 0.57$ and 0.60, respectively. Using this approach, we estimated the value of z of our $Sr_{1-x}La_xTi(^{16}O_{1-z}{}^{18}O_z)_3$ single-crystal samples.

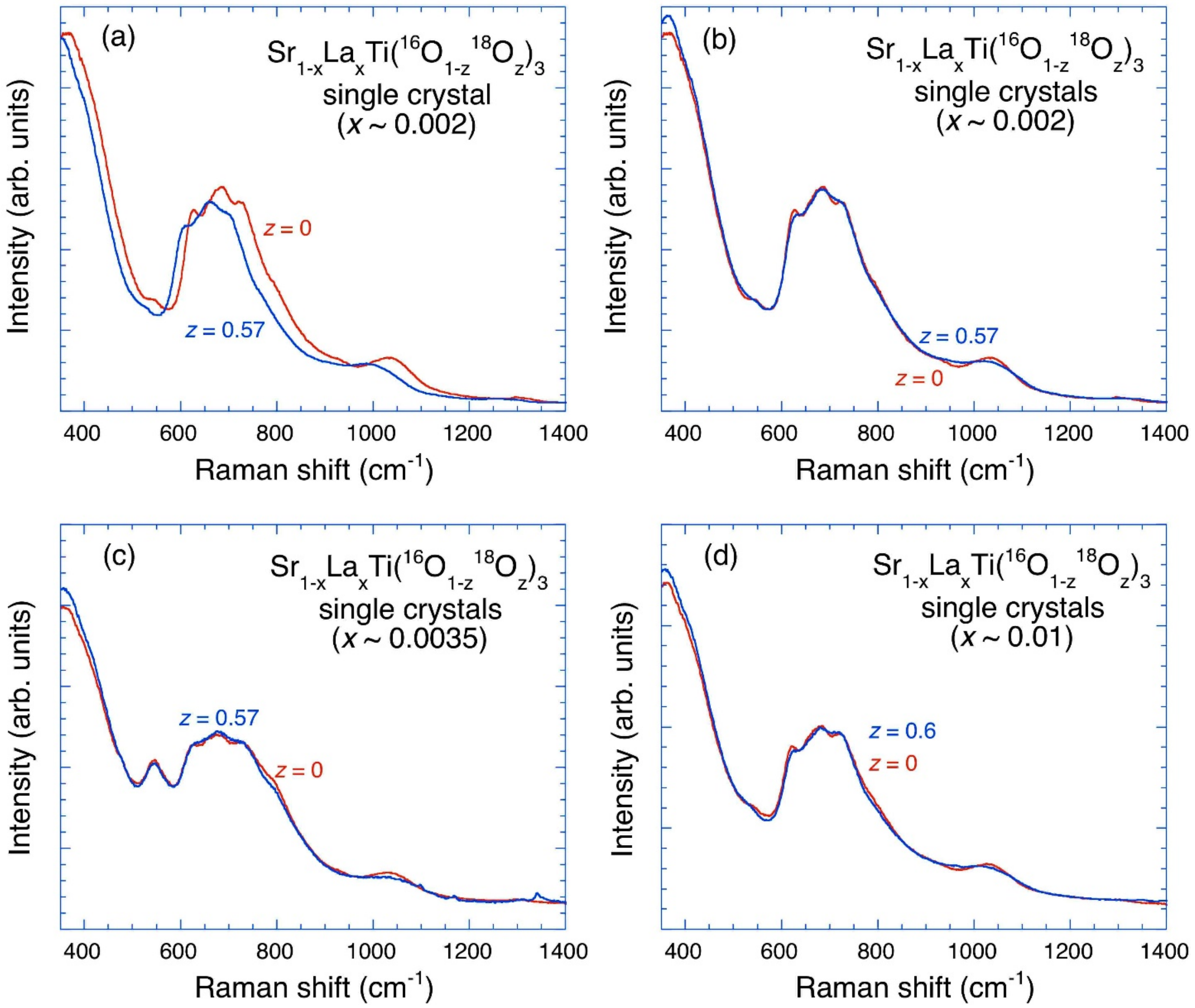

Supplementary Figure 1 | a, Raman scattering spectra plotted against frequency for both ^{18}O-free (x ~ 0.002 and z = 0) and ^{18}O-exchanged (x ~ 0.002 and z = 0.57) single crystals of $Sr_{1-x}La_xTi(^{16}O_{1-z}{}^{18}O_z)_3$ measured at room temperature. **b**, Same as **a** but the frequency for the ^{18}O-exchanged sample was multiplied by $[\{16(1-z) + 18z\}/16]^{1/2} = 1.035$ (i.e., $z = 0.57$) to achieve the best coincidence of the two spectra. **c**, Raman spectra for x ~ 0.0035 sample. The frequency for the ^{18}O-exchanged sample was multiplied by 1.035, which gives $z = 0.57$. **d**, Raman spectra for x ~ 0.01 sample. The frequency for the ^{18}O-exchanged sample was multiplied by 1.037, which gives $z = 0.60$.

Supplementary Note 2: Confirmation of the oxygen isotope (^{18}O) exchange ratio by the dynamic secondary ion mass spectrometry (SIMS)

In order to confirm the validity of the *z*-value estimation described above using the Raman spectra, we have also done the dynamic secondary ion mass spectrometry (SIMS) for our $Sr_{1-x}La_xTi(^{16}O_{1-z}{}^{18}O_z)_3$ ($x \sim 0.0035$ and $z = 0.57$) single-crystal sample. Here, the $z = 0.57$ was determined by the Raman spectroscopy. Dynamic SIMS measurements were performed by a scientific services company EAG Laboratories™ (www.eag.com). They use Cs ion beam to sputter material (so-called secondary ions are produced) from the surface of the sample, and a flat-bottomed crater is created on the sample surface. While scanning the surface by the ion beam, the secondary ions such as $^{18}O^{2-}$ and $^{16}O^{2-}$ only from the centre of the crater are acquired using a mass analyser. The depth scale was calibrated by measuring the crater depth using a stylus

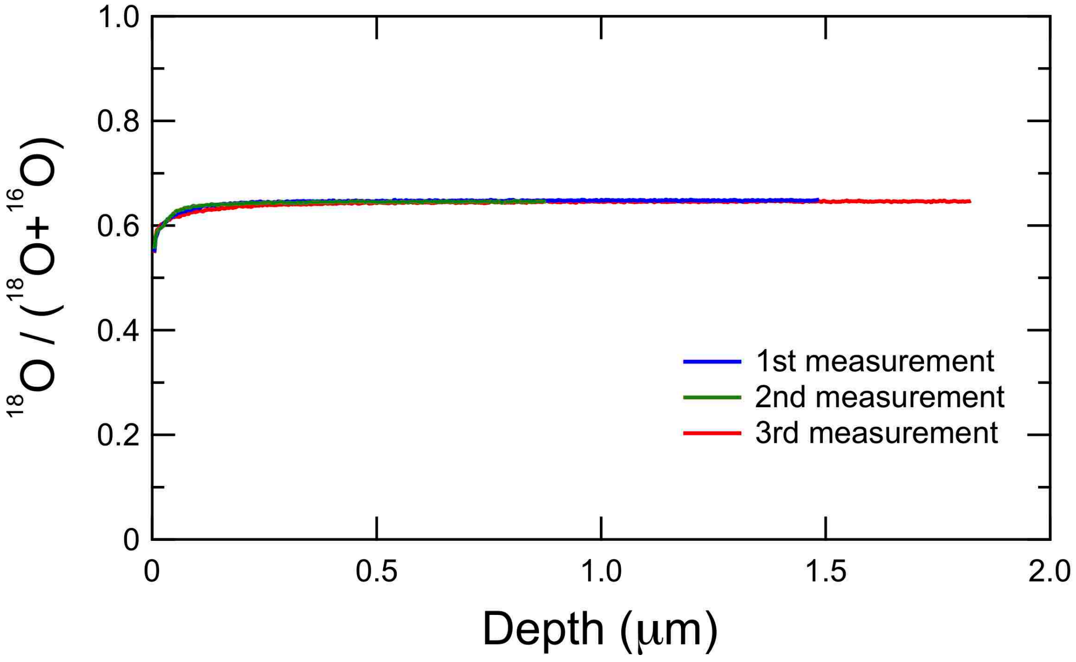

Supplementary Figure 2 | Depth profile of the concentration ratio of ^{18}O and ^{16}O atoms in our $Sr_{1-x}La_xTi(^{16}O_{1-z}{}^{18}O_z)_3$ single crystal ($x \sim 0.0035$) measured by the dynamic secondary ion mass spectrometry (SIMS). Three measurements gave almost identical results as $z = {}^{18}O / ({}^{16}O + {}^{18}O) \sim 0.65$.

profilometer. Then, the depth profile of the secondary ion signal is obtained. In general, the signal intensity of each secondary ion is linearly related to the concentration of the material. The proportionality coefficients are derived from the analysis of reference samples. For this purpose, we provided our $Sr_{1-x}La_xTi(^{16}O_{1-z}{}^{18}O_z)_3$ ($x \sim 0.0035$ and $z = 0$) single-crystal sample as well.

The results are shown in Supplementary Figure 2. The measurements were performed three times in different position of the sample surface; reproducibility of the data were extremely good. The quantitative accuracy of the secondary ion concentration is ±10-20%. Then, the obtained z value by this dynamic SIMS measurement is expressed as $z = 0.65 \pm 0.13$. This means the value we have deduced from the Raman spectra ($z = 0.57$) is in reasonable agreement. In the main text, we do not discuss anything depending on the precise value of z. Therefore, we can safely use the z value deduced by the Raman spectroscopy in the main text.

Supplementary Note 3: General trend of the mobility vs. carrier density: comparison with the literature

In Supplementary Reference 2, Behnia proposed an intriguing phenomenological model for the mobility of electron-doped $SrTiO_3$ systems as a function of the carrier density. The model seems to explain sufficiently the carrier mobility (most of the cases were the Hall mobility) of $SrTi_{1-x}Nb_xO_3$ and $SrTiO_{3-\delta}$ systems in the literature [3-6] at low temperatures around 4 K (see the data points in Supplementary Figure 3). It is quite surprising that, although the model neglected the evolution of both the effective mass and the effective Bohr radius for the large change of carrier density in four orders of magnitude, the fit of the model to the experimental results is quite satisfactory. The only parameter of the model is a kind of roughness of the dopant-potential distribution. The Nb substitution for Ti introduces a single electron in the centre of the TiO_2 octahedron while the oxygen deficiency introduces two electrons and distorts two neighbouring octahedra. Thus, the mobility for the $SrTi_{1-x}Nb_xO_3$ systems is about three times larger than that of the $SrTiO_{3-\delta}$ systems (see orange and green lines in Supplementary Figure 3).

The mobility of our $Sr_{1-x}La_xTiO_3$ single crystals is located between the two lines. We may say it is rather closer to the green line for the $SrTiO_{3-\delta}$ system. Moos et al. investigated the mobility of $Sr_{1-x}La_xTiO_3$ intensively [7], and concluded that the amount of oxygen deficiency is negligible and the carriers are dominantly provided by La^{3+}, but the mobility vs. carrier density relation for $SrTiO_{3-\delta}$ can be also applied to $Sr_{1-x}La_xTiO_3$ down to 19 K. In Supplementary Figure 3, we have plotted it for 4 K by the purple solid line. If we consider the other data of the $Sr_{1-x}La_xTiO_3$ systems [8, 9], it is difficult to say the model in Supplementary Reference 7 may fit well to the experimental data.

For more proper discussion on the mobility as a function of the carrier density, we need

further elaborate experiments with much more delicate control of the sample quality, dopants distribution, and its density. This is beyond the scope of our present work.

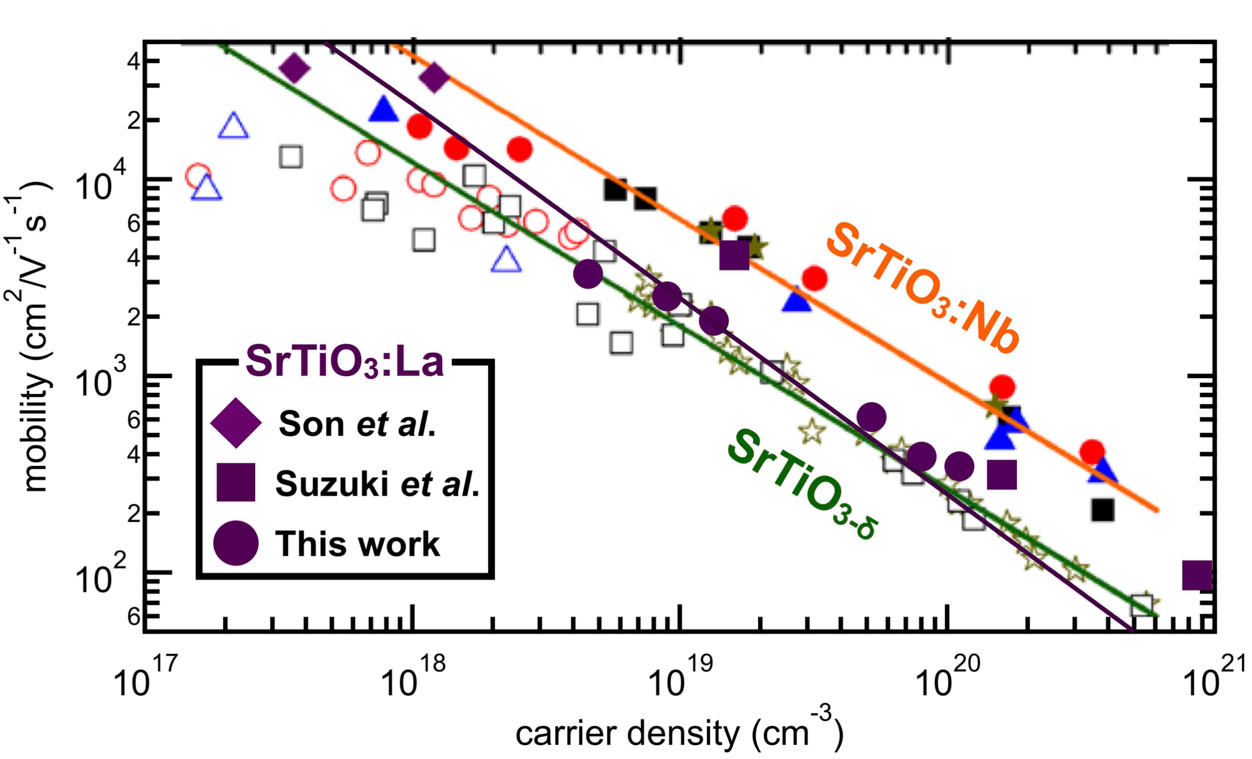

Supplementary Figure 3 | Mobility as a function of carrier density for electron-doped $SrTiO_3$ systems exhibited in Supplementary Reference 2. The data points were taken from the literature: $SrTi_{1-x}Nb_xO_3$ (red filled circles [3], black filled squares [4], green filled stars [5] and blue filled triangles [6]), $Sr_{1-x}La_xTiO_3$ (purple filled diamonds [8] and purple filled squares [9]), as well as $SrTiO_{3-\delta}$ (red opaque circles [3], black opaque squares [4], green opaque stars [5], blue opaque triangles [6]). Our $Sr_{1-x}La_xTiO_3$ (this work) corresponds to the purple filled circles. Solid lines represent the phenomenological estimation of the mobility vs. carrier-density relationships for $SrTi_{1-x}Nb_xO_3$ (orange) and $SrTiO_{3-\delta}$ (green) according to Supplementary Reference 2, and that for $Sr_{1-x}La_xTiO_3$ (purple) [7].

Supplementary Note 4: Superconducting transition for z ~ 0.4 single crystals of $Sr_{1-x}La_xTi(^{16}O_{1-z}{}^{18}O_z)_3$ (x ~ 0.001 and x ~ 0.005)

The single crystals of $Sr_{1-x}La_xTi(^{16}O_{1-z}{}^{18}O_z)_3$ (x ~ 0.001, z = 0.41, and x ~ 0.005, z = 0.37) were prepared as described in the main text (see Methods). The values of z were determined from Raman scattering measurements, as described above. The value of x is the nominal one. In Supplementary Figure 4, the resistivity of the two samples are plotted against temperature.

The inset of Supplementary Figure 4 shows the resistivity below 1 K. In this study, the superconducting critical temperature T_c is defined by the three dotted lines in the figure; the first line is almost equal to the residual resistivity before the resistivity drop, the second line represents the slope of the resistivity drop for the superconductivity, and the third line corresponds to the average resistivity of the stable almost-zero resistance state after the resistivity drop. The intersection of the first and the second lines gives the onset temperature of the superconductivity, whereas the intersection of the second and the third lines gives the end temperature of the superconductivity. We defined our T_c is the mid-point of the onset and end temperatures. Using this method, the values of T_c for the z ~ 0.4 samples were determined to be 0.41 K and 0.43 K for x ~ 0.001 and x ~ 0.005, respectively.

The zero-resistance state sometimes exhibited a small positive or negative offset, but it was due to the common-mode leakthrough caused by unequal contact resistances at the two voltage electrodes and has no effect on the above-defined T_c's.

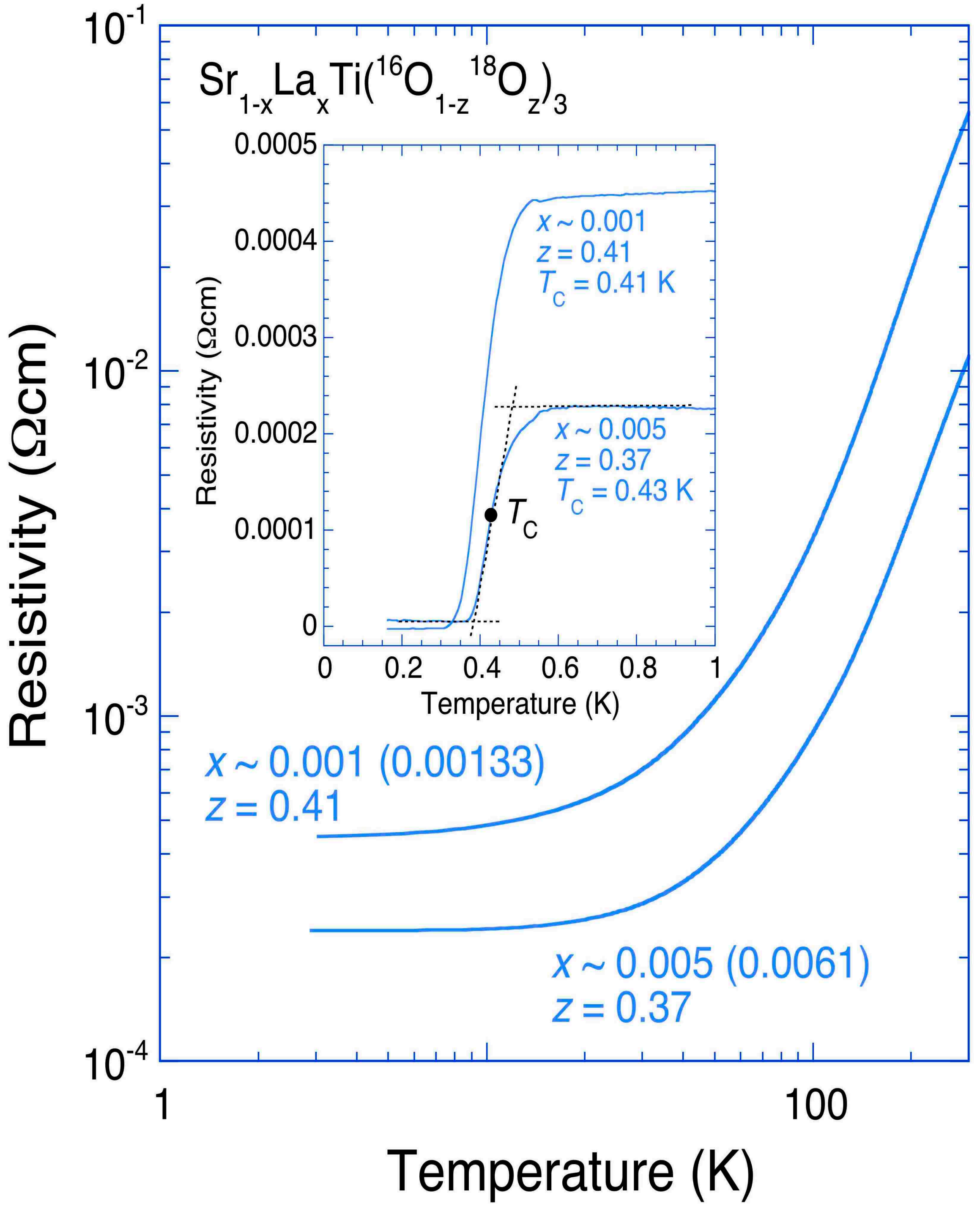

Supplementary Figure 4 | Temperature dependence of the resistivity of single crystals of $Sr_{1-x}La_xTi(^{16}O_{1-z}{}^{18}O_z)_3$ (x ~ 0.001, z = 0.41 and x ~ 0.005, z = 0.37). The inset shows the resistivity below 1 K. In this study, we defined T_c as a mid-point of the resistance drop as indicated by the three dotted lines in the inset. The values of T_c were 0.41 K and 0.43 K for x ~ 0.001 and x ~ 0.005, respectively. The numbers in the parentheses indicate the carrier numbers per a Ti atom.

Supplementary Note 5: Inductively determined transition temperature

The transition to the superconducting phase observed from the a.c. susceptibility (mutual inductance) measurements of $Sr_{1-x}La_xTi(^{16}O_{1-z}{}^{18}O_z)_3$ ($x \sim 0.0035$, $z = 0.57$) is plotted in Supplementary Figure 5. For the measurements, we have put wires of a set of induction and detection coils directly on the single crystals. The diameter/length of the induction (detection) coil were 3 mm / 11 mm (2 mm / 6 mm). The a.c. magnetic field was generated by injecting the induction coil with an excitation current of 250–500 μA, and the detection coil voltage was measured. The amplitude of the a.c. magnetic field was estimated to be approximately 0.025–0.05 Oe. The frequency was set at 15.9 Hz.

The transition temperature is defined as the onset of the voltage drop (~0.4 K), which is lower than the resistive $T_c \sim 0.55$ K. We used a superconducting wire for the coils to avoid a possible temperature rise due to the Joule heating of the coils, thus we believe this discrepancy is not a simple artefact of our experiment. Actually. this large discrepancy has been commonly observed in the superconductivity of $SrTiO_3$ in the literature (e.g., Supplementary References 5, 10, 11, and 12). Recently, it was proposed that the twin boundaries between tetragonal domains of $SrTiO_3$ are polar [13,14] with larger conductivity, resulting in a higher critical temperature [15,16]. This discrepancy indicates that there are some important factors that require future detailed measurements for clarification. Putting aside this issue, we used the resistive T_c in this study for fair comparison with the values of T_c in the literature.

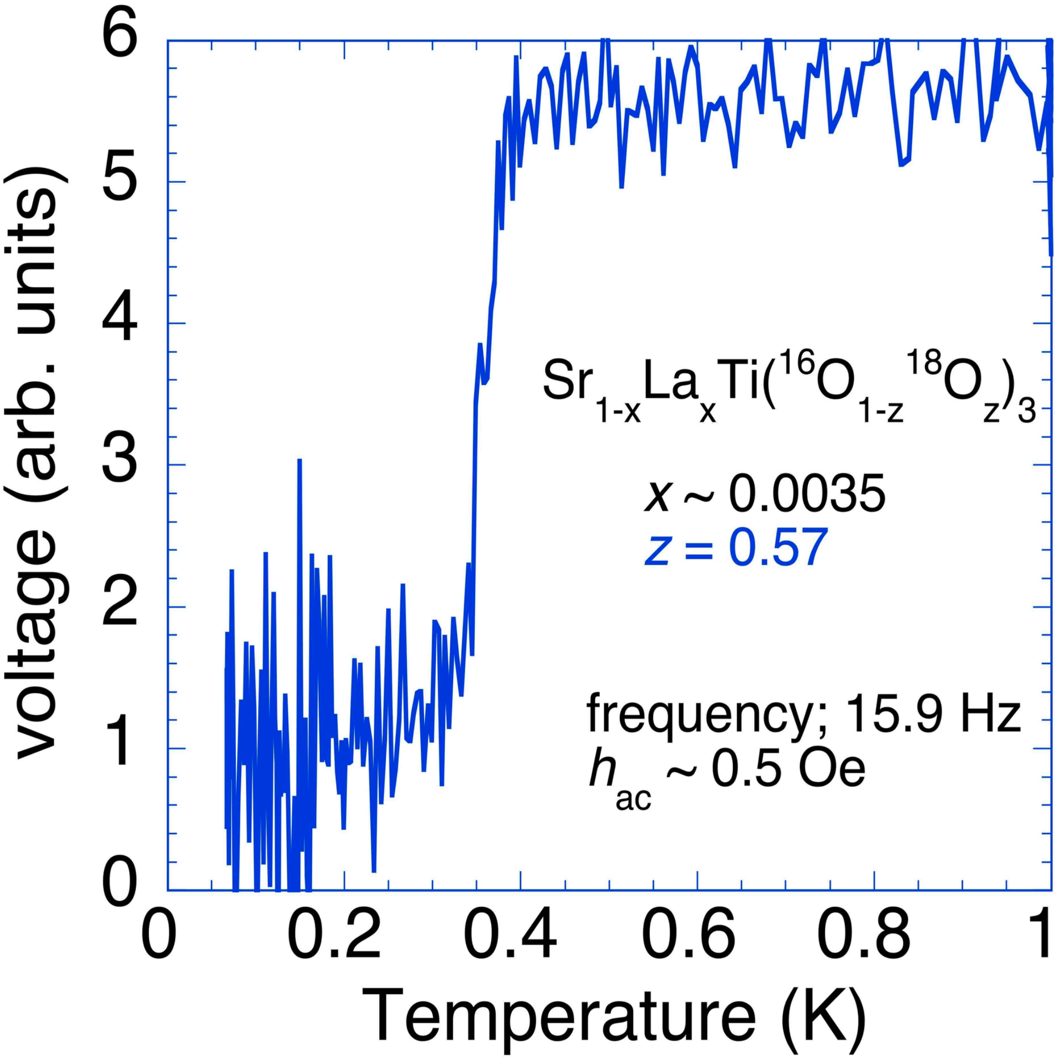

Supplementary Figure 5 | Temperature dependence of the detection coil voltage of the mutual inductance measurements for a single crystal of $Sr_{1-x}La_xTi(^{16}O_{1-z}{}^{18}O_z)_3$ (x ~ 0.0035, z = 0.57) below 1 K. The values of T_c defined as the onset of the voltage drop is ~0.4 K, which is smaller than the

Supplementary Note 6: Comparison of our $Sr_{1-x}La_xTiO_3$ system to $Sr_{1-x}Ca_xTiO_{3-\delta}$ and $Sr_{1-x}Ba_xTiO_{3-\delta}$

The $Sr_{1-x}Ca_xTiO_3$ shows symmetry-lowering antiferrodistortive (AFD) rotation of the TiO_6 octahedra because the ionic radius of Ca^{2+} is smaller than that of Sr^{2+}. The AFD rotation destroys the transition to a ferroelectric state [17–21]. In fact, the $Sr_{1-x}Ba_xTiO_3$ of $x = 0.006$, which does not show the AFD rotation undergoes a ferroelectric transition at approximately 15 K (Supplementary Reference 22). However, the $Sr_{1-x}Ca_xTiO_3$ system with the AFD rotation exhibits both ferroelectricity [16, 23–25] and superconductivity with carrier doping [10,16] only for a very small value of x ($\lesssim 0.016$) region. Moreover, there is a classic report [26] on polycrystalline $Sr_{1-x}Ba_xTiO_{3-\delta}$, where $0.025 \leq x \leq 0.125$ in the region of robust ferroelectricity, showing superconductivity with a maximum T_c of 0.52 K with carrier doping. Therefore, we think the appearance of ferroelectricity in this very small x region for both systems is not directly related to AFD; instead, the suppression of the ferroelectricity due to the large quantum fluctuation may be reduced by the Ca and Ba doping as an impurity.

As an analogue to $Sr_{1-x}Ba_xTiO_3$ and $Sr_{1-x}Ca_xTiO_3$ systems, we can reasonably assume that the $Sr_{1-x}La_xTiO_3$ system should have a ferroelectric QCP. There are some traditional arguments that ferroelectricity suppresses the superconductivity (see for example Supplementary Reference 27). We think that it may be rather difficult for our $Sr_{1-x}La_xTiO_3$ system to stand on such a viewpoint because our experimental data suggest that as a system approaches ferroelectricity, the value of T_c is enhanced (see main text). Recent theoretical investigations [28–30] have proposed the importance of the QCP. A phonon frequency related to the symmetry breaking structural phase transition vanishes at the QCP, and this phonon mode is responsible for the superconductivity. Therefore, we attempted to elucidate the effect of the QCP in the

$Sr_{1-x}La_xTiO_3$ system. However, the QCP of the system is hidden by the metallicity. Thus, it was necessary to estimate the location of the hidden QCP of the $Sr_{1-x}La_xTiO_3$ system.

For this purpose, we plotted the ferroelectric transition temperature T_{FE} as a function of the Ca or Ba substitution in Supplementary Figure 6. We used the data reported in the literature for $Sr_{1-x}Ba_xTiO_3$ [22,31] and $Sr_{1-x}Ca_xTiO_3$ [23]. (The region for $x \geq 0.16$ for $Sr_{1-x}Ca_xTiO_3$ is considered to be a relaxor ferroelectric [24, 25].) The data are expected to be compared with the empirical relation for a quantum ferroelectric $T_{FE} \sim (x - x_0)^{\beta}$, where x_0 corresponds to the QCP and $\beta = 0.5$, 0.25, or other values [32]. We tried to fit the experimental data of $Sr_{1-x}Ca_xTiO_3$ in the ferroelectric region ($0.002 \leq x \leq 0.02$) using $T_{FE} \sim A(x - x_0)^{0.5}$, as indicated by the blue dotted line in Supplementary Figure 6, and obtained $x_0 = 0.00022$. For $Sr_{1-x}Ba_xTiO_3$, the three data points were fitted by the $T_{FE} \sim x^{0.64}$ relation, as indicated by the red dotted line in Supplementary Figure 6. If we assume that the quantum fluctuation effect is approximately 1 K, a rough estimation of the QCP, i.e., the value of x_0, would be on the order of 0.0001. Then, we assumed that the QCP of $Sr_{1-x}La_xTiO_3$ may also be located near $x \sim 0.0001$. For the schematic discussion in the main text, this level of back-of-the-envelope estimation is sufficient.

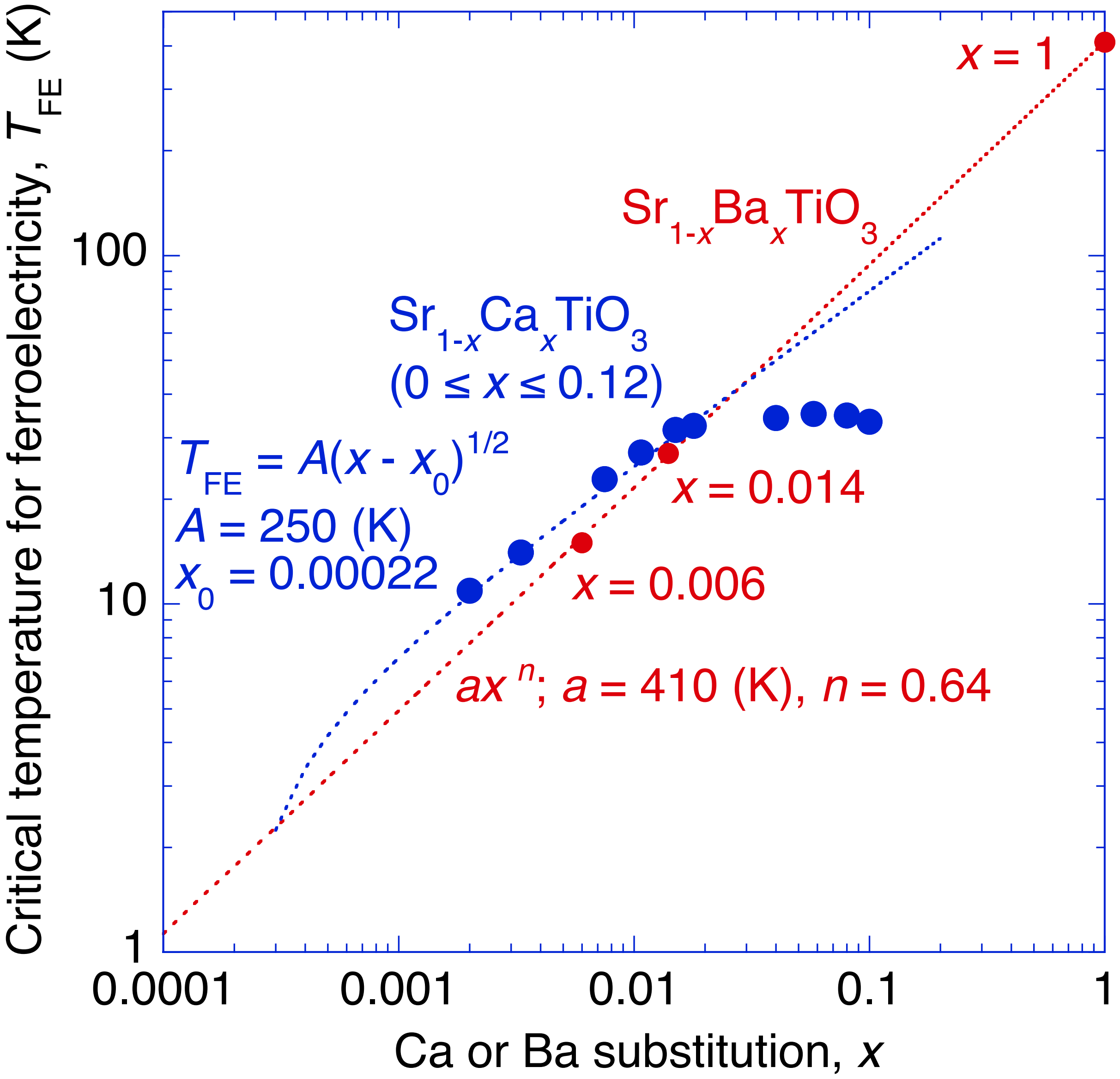

Supplementary Figure 6 | Critical temperatures of the ferroelectric (or relaxor ferroelectric) transition are plotted for $Sr_{1-x}Ca_xTiO_3$ (blue circles) and $Sr_{1-x}Ba_xTiO_3$ (red circles) against the Ca or Ba substitution on a logarithmic scale. The data were reported in the literature [22,23,31]. The $Sr_{1-x}Ca_xTiO_3$ data in the ferroelectric region ($x \leq 0.12$) are fitted by an empirical equation $T_{FE} \sim A(x - x_0)^{0.5}$ for the quantum ferroelectric transition (blue dotted line). The three data points of $Sr_{1-x}Ba_xTiO_3$ are all on the line of $T_{FE} \sim x^{0.64}$.

Supplementary Note 7: Trial to estimate the QCP from experimental data

Edge et al. proposed a phenomenological model [28] to describe the superconducting dome. They used a model for the ferroelectric fluctuation in analogy with that of magnetic phase transitions. In the model, the total energy is expressed by a double-well potential, where Γ is the tunnelling energy between the wells. The ferroelectric phase transition occurs at $\Gamma \sim 2J$, where J is roughly a nearest-neighbour coupling. They assumed $2J = 1$ for simplicity, such that the phonon frequency of the wave vector $\boldsymbol{q}$ is given as $\omega_{\boldsymbol{q}}^2 = 4\Gamma(\Gamma - \cos(q))$. The softening of the phonon (at the zone centre $\boldsymbol{q} = \boldsymbol{0}$), where the ferroelectric phase transition occurs, corresponds to $\omega_{\boldsymbol{q}=\boldsymbol{0}} \rightarrow 0$, which happens when $\Gamma \rightarrow \cos(0) = 1$. The McMillan formula of the Eliashberg theory in the strong-coupling limit gives the superconducting coupling constant λ as

$$\lambda = \int d\omega \frac{\alpha(\omega)^2 F(\omega)}{\omega} \xrightarrow{\text{v.H.s.}} \alpha^2 \int_{-\pi}^{\pi} \frac{dq}{2\sqrt{\Gamma(\Gamma - \cos q)}} \quad \ldots..(1)$$

under the limit of the van Hove singularity at $\omega = \omega_{\boldsymbol{q}}$. Here, α represents the electron–phonon coupling constant and $F(\omega)$ is the spectral density of phonons. Supplementary Equation 1 gives the relationship between λ and Γ.

On the other hand, by solving the standard gap equation, the relationship between λ and T_c can be obtained [33]:

$$\frac{D}{\lambda} = \sqrt{T_c} \int_{-E_F/T_c}^{0} dx \sqrt{x + E_F/T_c} \, \frac{\tanh(x/2)}{x} \ldots..(2)$$

D is not a parameter but a constant of 190 $K^{1/2}$, and E_F is the Fermi energy proportional to $n^{2/3}$, where n is the carrier density. Using Supplementary Equation 2, we calculated the values of λ for our $Sr_{1-x}La_xTiO_3$ ($0.0005 \leq x \leq 0.01$) and $Sr_{1-x}La_xTi(^{16}O_{1-z}{}^{18}O_z)_3$ ($z = 0.57$ and 0.60; $x \sim 0.002$,

0.0035, and 0.01) with the experimentally obtained n (for E_F) and T_c listed in Tables 1 and 2 in the main text. Then, by numerically solving Supplementary Equation 1, we obtained the value of Γ/α for an arbitrary value of α. The softening occurs when $\Gamma/\alpha \rightarrow 1/\alpha$ regardless of the value of α. Because we are not interested in the exact value of Γ/α but on the softening ($\Gamma \rightarrow 1$), we can safely assume $\alpha = 1$ for the discussion here. All the results are listed in Supplementary Table 1. (For the data in the literature, i.e., for $SrTiO_{3-\delta}$, we similarly calculated the values of λ using the values of n and T_c in Supplementary Reference 12 and deduced the values of Γ as discussed above.)

In Fig. 3**c** and 3**d** in the main text, all the values of Γ are plotted as a function of the Fermi temperature T_F. In the range of T_F of the experimental data sets, the values of Γ were all larger than one. All of our $Sr_{1-x}La_xTiO_3$ single crystals were in the region of 50 K $\lesssim T_F \lesssim$ 450 K, which was estimated from n using a naive free-electron model with triply degenerated Ti 3d t_{2g} bands. $T_F = E_F/k_B$ scales with $n^{2/3}$, where k_B is the Boltzmann constant. The naive value of T_F may be modified a couple of times consistently for all the data because of the mass renormalisation or the fermiology but would not differ by more than an order of magnitude. Because the modification is proportional to T_F, we only need to re-scale the horizontal axis. Thus, the modification does not affect any of the results presented here. Edge et al. assumed that Γ would be given as a quadratic function of T_F, which means by increasing the carrier density (by increasing T_F), the tunnelling energy Γ must increase; otherwise, the ferroelectricity is not destroyed [28]. Therefore, we assumed $\Gamma = A + BT_F + CT_F^2$ following this scenario and attempted to fit this model to the Γ vs. T_F relation deduced from our experimental data $Sr_{1-x}La_xTiO_3$ ($0.0005 \leq x \leq 0.01$) as well as those in Supplementary Reference 12 ($SrTiO_{3-\delta}$). The solid lines in Fig. 3**c**

and 3**d** in the main text are the results of our fittings. As for $SrTiO_{3-\delta}$, we did another fitting by using only the larger carrier density region (dash-dotted line). The parameters A, B, and C are 0.92, 2.27 × 10^{-3}, and 2.63 × 10^{-6}, respectively, for the lower carrier density $SrTiO_{3-\delta}$, 0.83, 3.54 × 10^{-3}, and –3.95 × 10^{-7}, respectively, for the larger carrier density $SrTiO_{3-\delta}$, and 0.85, 2.92 × 10^{-3}, and 1.36 × 10^{-6}, respectively, for our $Sr_{1-x}La_xTiO_3$. All the lines fit fairly well to the values of Γ deduced from the experimental data. Using the same values of B and C for $Sr_{1-x}La_xTiO_3$, we also fit our $Sr_{1-x}La_xTi(^{16}O_{1-z}{}^{18}O_z)_3$ ($z \sim 0.6$). The value of the parameter A is 0.77.

Supplementary Table 1 | Coupling constant and tunnelling energy for $Sr_{1-x}La_xTiO_3$ single crystals

x	T_F (K)	λ	Γ
~0.0005	87.76	3.79	1.11
~0.001	114.77	3.23	1.20
~0.002	183.09	2.47	1.44
~0.003	283.78	1.86	1.80
~0.0035	307.61	1.80	1.86
~0.005	378.01	1.53	2.15
~0.007	469.86	1.30	2.50
~0.01	667.61	0.94	3.41

The nominal value of x, the Fermi temperature T_F deduced from the carrier density estimated from the Hall effect measurements, and the λ and Γ values calculated numerically using Supplementary Equations 1 and 2 for $Sr_{1-x}La_xTiO_3$ single crystals in this study.

Supplementary Note 8: Hall effect measurements

For all the crystals of $Sr_{1-x}La_xTi(^{16}O_{1-z}{}^{18}O_z)_3$ investigated in this study, the Hall coefficient R_H deduced from the slope of the Hall resistivity ρ_H plotted against an external magnetic field $\mu_0 H$ did not change for eight different temperatures of 5, 30, 50, 100, 150, 200, 250, and 300 K. Here, μ_0 represents the permeability of the vacuum.

Supplementary Figures 7(a) and 7(b) show the ρ_H vs. $\mu_0 H$ relation at 5 K for $Sr_{1-x}La_xTiO_3$ single crystals with $x \sim 0.0003$, 0.0005, and 0.001 (a) as well as with $x \sim 0.003$, 0.005, and 0.007 (b). The resistivity vs. temperature relations of the same samples are presented in Fig. 1**a** in the main text. For each single crystal, the temperature-independent carrier density n was calculated using $R_H = e^{-1}n^{-1}$, where e represents the elementary charge. From the obtained values of n, we calculated the values of x that are indicated for each ρ_H vs. $\mu_0 H$ relation. The results are presented in Table 1 and Table 2 of the main text.

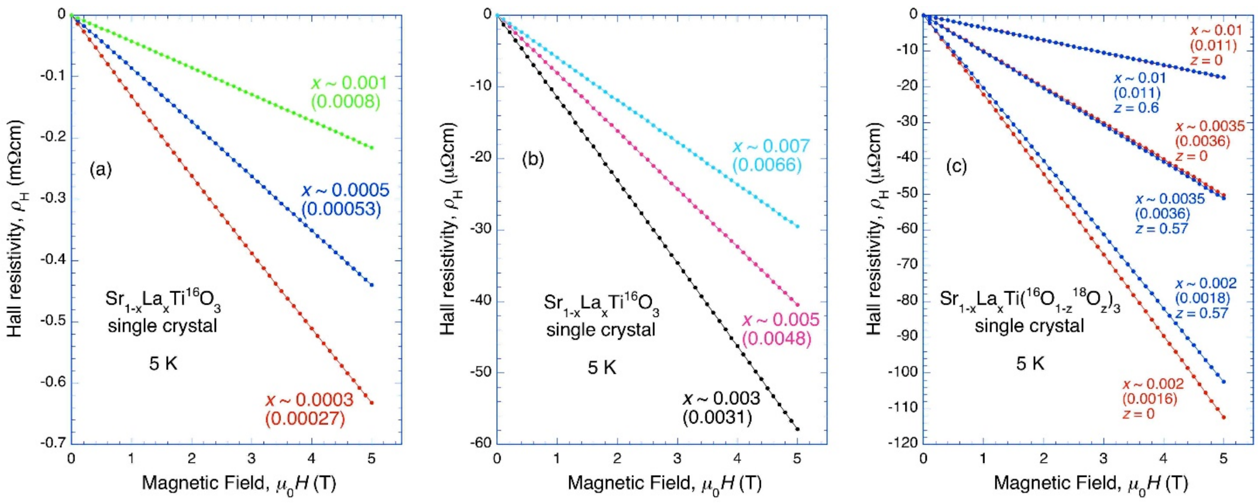

Supplementary Figure 7 | a, Hall resistivity ρ_H plotted against external magnetic field $\mu_0 H$ at 5 K for $Sr_{1-x}La_xTiO_3$ single crystals with $x \sim 0.0003$, 0.0005, and 0.001. **b,** ρ_H plotted against $\mu_0 H$ at 5 K for $Sr_{1-x}La_xTiO_3$ single crystals with $x \sim 0.003$, 0.005, and 0.007. Note that the vertical scale differs from that in **a** because of the difference in the carrier density. **c,** ρ_H plotted against $\mu_0 H$ at 5 K for $Sr_{1-x}La_xTi(^{16}O_{1-z}{}^{18}O_z)_3$ with (x, z) = (~0.002, 0), (~0.002, 0.57), (~0.0035, 0), (~0.0035, 0.57), (~0.01, 0), and (~0.01, 0.60). Each numerical value in parentheses corresponds to the number of electrons per Ti site deduced from this Hall effect measurement. These values agree well with the nominal value of x.

Supplementary Figure 7(c) shows the ρ_H at 5 K for the $z = 0$ and ^{18}O-exchanged single crystals of $Sr_{1-x}La_xTi(^{16}O_{1-z}{}^{18}O_z)_3$ with (x, z) = (~0.002, 0), (~0.002, 0.57), (~0.0035, 0), (~0.0035, 0.57), (~0.01, 0), and (~0.01, 0.60). The temperature dependences of the resistivity of the same samples are presented in Fig. 2**a** in the main text. The values of x were deduced from these ρ_H vs. $\mu_0 H$ relations.

Supplementary References

[1] Nilsen, W. G. & Skinner, J. G. Raman spectrum of Strontium Titanate. *J. Chem. Phys.* **48**, 2240 (1968).

[2] Behnia, K. On mobility of electrons in a shallow Fermi sea over a rough seafloor. *J. Phys.: Condens. Matter* **27**, 375501 (2015).

[3] Lin, X., Bridoux, G., Gourgout, A., Seyfarth. G., Krämer, S., Nardone, M., Fauque, B. & Behnia, K. Critical Doping for the Onset of a Two-Band Superconducting Ground State in $SrTiO_{3-\delta}$. *Phys. Rev. Lett.* **112**, 207002 (2014).

[4] Frederikse, H. P. R. & Hosler, W. R. Hall mobility in $SrTiO_3$. Phys. Rev. **161**, 822 (1967).

[5] Koonce, C. S., Cohen, M. L., Schooley, J. F., Hosler, W. R., & Pfeiffer, E. R. Superconducting Transition Temperatures of Semiconducting $SrTiO_3$. *Phys. Rev.* **163**, 380-390 (1967).

[6] Spinelli, A., Torija, M. A., Liu, C., Jan, C. & Leighton, C. Electronic transport in doped $SrTiO_3$: Conduction mechanisms and potential applications. *Phys. Rev. B* **81**, 155110 (2010).

[7] Moos, R. & Härdtl, K. H. Electronic transport properties of $Sr_{1-x}La_xTiO_3$ ceramics. *J. Appl. Phys.* **80**, 393 (1996).

[8] Son, J., Moetakef, P., Jalan, B., Bierwagen, O., Wright, N. J., Engel-Herbert, R. & Stemmer, S. Epitaxial $SrTiO_3$ films with electron mobilities exceeding 30,000 $cm^2V^{-1}s^{-1}$. *Nat. Mater.* **9**, 482 (2010).

[9] Suzuki, H., Bando, H., Ootsuka, Y., Inoue, I. H., Yamamoto, T., Takahashi, K. & Nishihara, Y. Superconductivity in Single-Crystalline $Sr_{1-x}La_xTiO_3$. *J. Phys. Soc. Jpn.* **65**, 1529-1532 (1996).

[10] Rischau, C. W., Lin, X., Grams, C. P., Finck, D., Harms, S., Engelmayer, J., Lorentz, T., Gallais, Y., Fauque, B., Hemberger, J. & Behnia, K. A ferroelectric quantum phase transition inside the superconducting dome of $Sr_{1-x}Ca_xTiO_{3-\delta}$. *Nat. Phys.* **13**, 643-648 (2017).

[11] Stucky, A., Scheerer, G. W., Ren, Z., Jaccard, D., Poumirol, J.-M., Barreteau, C., Giannini, E. & van der Marel, D. Isotope effect in superconducting n-doped $SrTiO_3$. *Sci. Rep.* **6**, 37582 (2016).

[12] Schooley, J. F., Hosler, W. R., Ambler, E., Becker, J. H., Cohen, M. L. & Koonce C. S. Dependence of the superconducting transition temperature on carrier concentration in semiconducting $SrTiO_3$. *Phys. Rev. Lett.* **14**, 305 (1965).

[13] Salje, E. K. H., Aktas, O., Carpenter, M. A., Laguta, V. V. & Scott, J. F. Domains within domains and walls within walls: evidence for polar domains in cryongenic $SrTiO_3$. *Phys. Rev. Lett.* **111**, 247603 (2013).

[14] Ma, H. J. H., Scharinger, S., Zeng, S. W., Kohlberger, D., Lange, M., Stohr, A., Wang, X. R., Venkatesan, T., Kleiner, R., Scott, J. F., Coey, J. M. D., Koelle, D. & Ariando. Local Electrical Imaging of Tetragonal Domains and Field-Induced Ferroelectric Twin Walls in Conducting $SrTiO_3$. *Phys. Rev. Lett.* **116**, 257601 (2016).

[15] Lin, X., Gourgout, A., Bridoux, G., Jomard, F., Pourret, A., Fauqué, B., Aoki, D. & Behnia, K. Multiple nodeless superconducting gaps in optimally doped $SrTi_{1-x}Nb_xO_3$. *Phys. Rev. B* **90**, 140508(R) (2014).

[16] de Lima, B. S., da Luz, M. S., Oliveira, F. S., Alves, L. M. S., dos Santos, C. A. M., Jomard, F., Sidis, Y., Bourges, P., Harms, S., Grams, C. P., Hemberger, J., Lin, X., Fauqué, B. & Behnia, K. Interplay between antiferrodistortive, ferroelectric, and superconducting instabilities in $Sr_{1-x}Ca_xTiO_{3-\delta}$. *Phys. Rev. B* **91**, 045108 (2015).

[17] Vogt, H. Refined treatment of the model of linearly coupled anharmonic oscillators and its application to the temperature dependence of the zone-center soft-mode frequencies of $KTaO_3$ and $SrTiO_3$. *Phys. Rev. B* **51**, 8046 (1995).

[18] Belokopytov, G. V. Low-temperature nonlinear microwave response of incipient ferroelectrics. *Ferroelectrics* **168**, 69 (1995).

[19] Yamanaka, A., Kataoka, M., Inaba, Y., Inoue, K., Hehlen, B. & Courtens, E. Evidence for competing orderings in strontium titanate from hyper-Raman scattering spectroscopy. *Europhys. Lett*. **50**, 688 (2000).

[20] Aschauer U. & Spaldin N. Competition and cooperation between antiferrodistortive and ferroelectric instabilities in the model perovskite $SrTiO_3$. *J. Phys.: Condens. Matter* **26**, 122203 (2014).

[21] Zhong, W. & Vanderbilt, D. Competing Structural Instabilities in Cubic Perovskites. *Phys. Rev. Lett.* **74**,2587 (1995).

[22] Miura, S., Marutake, M., Unoki, H., Uwe, H. & Sakudo, T. Composition Dependence of the Phase Transition Temperatures in the Mixed Crystal Systems near $SrTiO_3$. *J. Phys. Soc. Jpn.* **38**, 1056-1060 (1975).

[23] Bednorz, J. G. & Müller, K. A. $Sr_{1-x}Ca_xTiO_3$: an *XY* quantum ferroelectric with transition to randomness. *Phys. Rev. Lett.* **52**, 2289-2292 (1984).

[24] Ranjan, R., Pandey, D. & Lalla, N. P. Novel features of $Sr_{1-x}Ca_xTiO_3$ phase diagram: evidence for competing antiferroelectric and ferroelectric interactions. *Phys. Rev. Lett.* **84**, 3726 (2000).

[25] Wang, J.-X., Liu, M.-F., Yan, Z.-B. & Liu, J.-M. Critical exponents of ferroelectric transitions in modulated $SrTiO_3$: consequences of quantum fluctuations and quenched disorder. *Chin. Phys. B* **22**, 077701 (2013).

[26] Schooley, J. F., Frederikse, H. P. R., Hosler, W. R. & Pfeiffer, E. R. Superconductive Properties of Ceramic Mixed Titanates. *Phys. Rev.* **159**, 301 (1967).

[27] Krivolapov, Y., Mann, A. & Birman, Joseph L. Theory of coexistence of superconductivity and ferroelectricity. *Phys. Rev. B* **75**, 092503 (2007).

[28] Edge, J. M., Kedem, Y., Aschauer, U., Spaldin, N. A. & Balatsky, A. V. Quantum Critical

Origin of the Superconducting Dome in $SrTiO_3$. *Phys. Rev. Lett.* **115**, 247002 (2015).

[29] Kedem, Y., Zhu, J-X. & Balatsky, A. V. Unusual superconducting isotope effect in the presence of a quantum criticality. *Phys. Rev. B* **93**, 184507 (2016).

[30] Dunnett, K., Narayan, A., Spaldin, N. A. & Balatsky, A. V. Strain and ferroelectric soft-mode induced superconductivity in strontium titanate. *Phys. Rev. B* **97**, 144506 (2018).

[31] Lemanov, V. V., Smirnova, E. P., Syrnikov, P. P. & Tarakanov, E. A. Phase transitions and glasslike behavior in $Sr_{1-x}Ba_xTiO_3$. *Phys. Rev. B* **54**, 3151-3157 (1996).

[32] Tchouobiap, S E Mkam, A theoretical study of soft mode behavior and ferroelectric phase transition in ^{18}O-isotope exchange $SrTiO_3$: evidence of phase coexistence at the quantum critical point. *Phys. Scr.* **89**, 025702 (2014).

[33] van der Marel, D., van Mechelen, J. L. M., Mazin, I. I. Common Fermi-liquid origin of T^2 resistivity and superconductivity in n-type $SrTiO_3$. *Phys. Rev. B* **84**, 205111 (2011).
